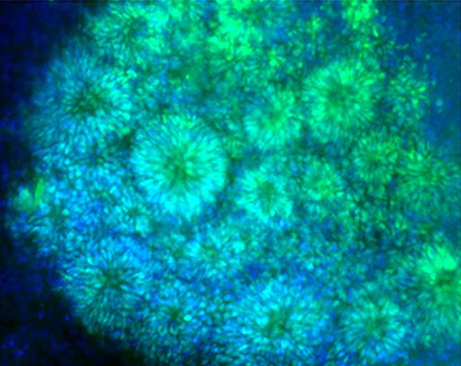# Modelling drug-induced neocortical toxicity *in vitro*

**Published:** 2014-12

The human cerebral cortex is affected by a wide range of disorders and is also particularly sensitive to the effects of drugs and environmental toxins during development. Human pluripotent stem cells (hPSCs) are a unique system to model the human cerebral cortex, but replicating the complexity of this structure in terms of both neuronal diversity and developmental processes has been limited in current models. Here, Chun-Ting Lee and colleagues use hPSC aggregates without cell dissociation but with inclusion of key trophic factors to model human neocortical development. They are able to reproduce *in vitro* the formation of radial glia scaffolding (which supports neuronal migration), of deep and upper layers of cortical neurons, and of different neuronal subtypes (including glutamatergic and GABAergic neurons), as occurs during human neocortical development. In addition, the authors use this system to study the effects of cocaine – a drug that causes developmental toxicity in humans – and find that it induces oxidative stress and impairs both neuronal differentiation and layer patterning. Notably, these changes are reversed by cimetidine, a cytochrome P450 inhibitor. This model represents a valuable system to study human neocortical development, its susceptibility to toxins and also to identify potential compounds to prevent drug-induced neocortical impairments. **Page 1397**

**Figure:**